# Cloning, Functional Characterization, and Catalytic Mechanism of a *Bergaptol O-Methyltransferase* from *Peucedanum praeruptorum* Dunn

**DOI:** 10.3389/fpls.2016.00722

**Published:** 2016-05-25

**Authors:** Yucheng Zhao, Nana Wang, Zhixiong Zeng, Sheng Xu, Chuanlong Huang, Wei Wang, Tingting Liu, Jun Luo, Lingyi Kong

**Affiliations:** ^1^State Key Laboratory of Natural Medicines, Department of Natural Medicinal Chemistry, China Pharmaceutical University, NanjingChina; ^2^College of Life Science and Technology, Huazhong Agricultural University, WuhanChina; ^3^Institute of Botany, Jiangsu Province and Chinese Academy of Sciences, NanjingChina

**Keywords:** *Peucedanum praeruptorum*, coumarins, *O-methyltransferase* (OMT), biosynthesis mechanisms, docking

## Abstract

Coumarins are main active components of *Peucedanum praeruptorum* Dunn. Among them, methoxylated coumarin compound, such as bergapten, xanthotoxin, and isopimpinellin, has high officinal value and plays an important role in medicinal field. However, major issues associated with the biosynthesis mechanism of coumarins remain unsolved and no corresponding enzyme has been cloned from *P. praeruptorum*. In this study, a local BLASTN program was conducted to find the candidate genes from *P. praeruptorum* transcriptome database using the nucleotide sequence of *Ammi majus bergaptol O-methyltransferase* (*AmBMT*, GenBank accession No: AY443006) as a template. As a result, a 1335 bp full-length of cDNA sequence which contains an open reading frame of 1080 bp encoding a BMT polypeptide of 359 amino acids was obtained. The recombinant protein was functionally expressed in *Escherichia coli* and displayed an observed activity to bergaptol. *In vitro* experiments show that the protein has narrow substrate specificity for bergaptol. Expression profile indicated that the cloned gene had a higher expression level in roots and can be induced by methyl jasmonate (MeJA). Subcellular localization analysis showed that the BMT protein was located in cytoplasm *in planta*. Homology modeling and docking based site-directed mutagenesis have been employed to investigate the amino acid residues in BMT required for substrate binding and catalysis. Conservative amino acid substitutions at residue H264 affected BMT catalysis, whereas substitutions at residues F171, M175, D226, and L312 affected substrate binding. The systemic study summarized here will enlarge our knowledge on OMTs and provide useful information in investigating the coumarins biosynthesis mechanism in *P. praeruptorum.*

## Introduction

Coumarins refer to the compound that has a 2H-1-benzopyran-2-one core structure. It is listed as one of the main components of plant secondary metabolites with widely distribution in the plant kingdom. As the main source of coumarins in Traditional Chinese Medicine, *Peucedanum praeruptorum* has been listed in current Pharmacopeia of the Peoples’ Republic of China with an application history of over 1500 years (Shao, 2010). It is employed as a kind of herbal medicine for reducing fevers and resolving phlegm. Besides, there are also examples that the extracts of *P. praeruptorum* display anti-cancer, anti-inflammatory, anti-hyperglycemic, anti-oxidant, and calcium-channel-blocking properties (Wu et al., 2003; Kumar et al., 2009; Yu et al., 2012; Zhou et al., 2014). However, as for coumarins from *P. praeruptorum*, the current work mainly focused on the extraction and activity analysis of the compound, little is known about the biosynthetic mechanism of coumarins in *P. praeruptorum* although plenty of work has been published in studying the biosynthesis mechanisms of coumarins in other species (Ling-Yi et al., 1996; Bourgaud et al., 2006; Karamat et al., 2014). For instance, angular pyranocoumarins, angular furanocoumarins, linear pyranocoumarins, linear furanocoumarins, and simple coumarins are all isolated from *P. praeruptorum* (among them, the angular products contribute to the main proportion of coumarins), however, only the biosynthesis mechanisms of simple coumarins were investigated (Ling-Yi et al., 1996; Bourgaud et al., 2006; Karamat et al., 2014; Zhao et al., 2015).

In recent years, next-generation sequencing technology (NGS) has emerged as a primary tool for high-through-put sequencing to discover unknown genes involved in plant secondary metabolism (Yonekura-Sakakibara et al., 2012; Li and Lan, 2015; Li et al., 2015). For instance, “guilt-by-association” principle and “co-expression analysis” are popular in new gene discovery with the help of NGS in the past 10 years (Saito et al., 2008; Usadel et al., 2009; Yonekura-Sakakibara et al., 2013). These methods display a good performance in those species that have genome sequence or at least one authenticated gene in the target pathway. However, it seems not straightforward in no-model plants especially those without reference conserved co-expression clusters identified (Saito et al., 2008; Usadel et al., 2009; Movahedi et al., 2012; Yonekura-Sakakibara et al., 2013). Thus, it is useless in coumarins biosynthesis studying of *P. praeruptorum* for no genes which have been functionally confirmed in coumarins biosynthesis so far. Although a transcriptome database of *P. praeruptorum* had been constructed within the years (Zhao et al., 2015), mountains of work need to be done to investigate the coumarins biosynthesis mechanism in *P. praeruptorum.*

**FIGURE 1:**
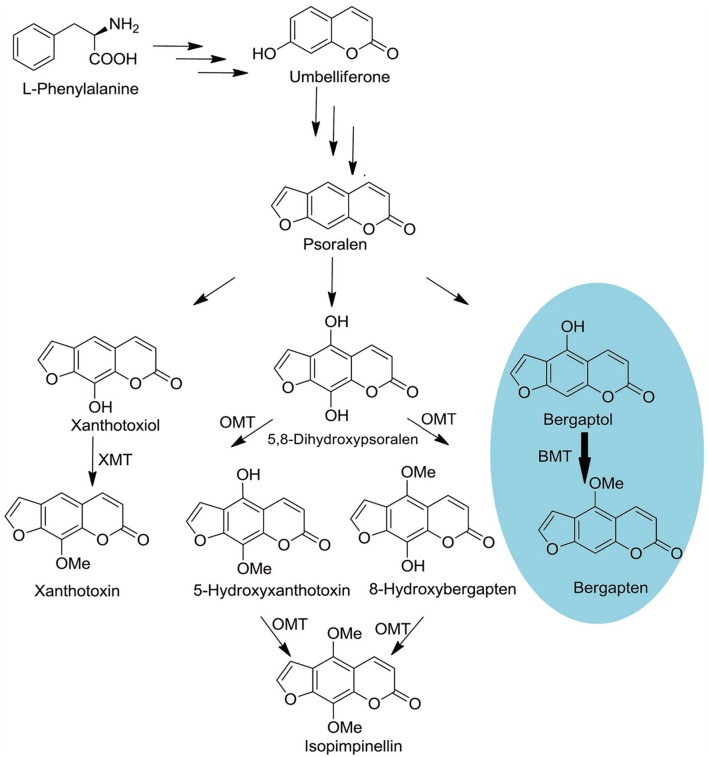
**Schematic outline of OMTs involved coumarins biosynthesis.** *O-MT*, *O-methyl-transferase; XMT, Xanthotoxiol O-methyltransferase; BMT, Bergaptol O-methyltransferase*. The oval in blue is the reaction investigated in this study.

As a universal process critical to all organisms, methylation of oxygen is also an important step in plant secondary metabolism (Zubieta et al., 2001). It not only participates in cell biological processes but also alters the solubility of the compounds and ultimately determines their special chemical and physical properties, namely synthesis of the lignin precursors, being used as pharmaceutically active substances and protection against UV photo-damage (Zubieta et al., 2001; Louie et al., 2010; Green et al., 2014). In *P. praeruptorum*, methylation by *S*-adenosyl-L-methionine (SAM) dependent *O*-methyltransferases (OMTs) is a common modification in coumarins biosynthesis, which creates a series of structure modified compounds (**Figure 1**) (Bourgaud et al., 2006; Zhao et al., 2015). However, some biosynthetic pathways of important compounds, especially the angular pyranocoumarins, are still unclear even in biochemical level (Bourgaud et al., 2006). Although the enzyme activities of some OMT have been biochemically characterized in the past 30 years, only the gene of *bergaptol O-methyltransferase* (BMT) has been cloned in recent years (Hehmann et al., 2004; Ishikawa et al., 2009; Lo et al., 2012). This situation largely impedes the investigation of coumarins biosynthesis mechanisms in *P. praeruptorum.*

In this study, we firstly report the cloning and biochemical characterization of a BMT cDNA from *P. praeruptorum*. The recombinant enzyme activity toward bergaptol and other substrates is demonstrated. The results show that the BMT has a rigid substrate affinity to bergaptol. In order to investigate the characters of the enzyme in plant, tissue-specific expression and subcellular localization were conducted. The results show that the BMT gene can be highly expressed in roots and is located in cytoplasm *in planta*. To investigate the catalytic mechanism of BMT, homology modeling and docking was conducted using alfalfa *caffeic acid/5-hydroxyferulic acid 3/5-O-methyltransferase* as the template (*MsCOMT*, PDB accession No: 1KYZ; Zubieta et al., 2002). Site-directed mutagenesis was subsequently proceeded to confirm the docking results. The experiment results indicated that F171, M175, D226, and L312 were critical to substrate binding while H264 were responsible for catalytic activity. To the best of our knowledge, the work reported here is the first investigation and identification of gene involved in coumarins biosynthesis in *P. praeruptorum.* The experiment and catalytic mechanism of BMT reported here will give more references insight into the mechanism and regulation of coumarins biosynthesis in *P. praeruptorum*.

## Materials and Methods

### Chemicals and Reagents

Unless otherwise stated, chemicals and reagents that used in this study were purchased from either Sigma–Aldrich (St. Louis, MO, USA) or Aladdin (Shanghai, China). Kanamycin was purchased from Melonepharm (Dalian, China). (*E*)-2′-hydroxychalcone and (*E*)-3′-hydroxychalcone were synthesized in our laboratory.

### Plant Material and cDNA Cloning

The 2-year-old *P. praeruptorum* material was collected from the fields of Ningguo City, Anhui Province, China. The whole plant was collected and immediately frozen and maintained in liquid nitrogen until use. The total RNA was isolated using TransZol Plant reagent (TransGen Biotech, Beijing, China) and used as a template for cDNA synthesis. The integrity and quality of total RNA were assessed by the ratio of 28S:18S RNA on a denaturing formaldehyde agarose gel by staining with EtBr. The cDNA with a ratio approximately 2:1 was used for experiment. First-Strand cDNA was prepared using total or poly A^+^ RNA according to the guides suggested in SMARTer^TM^ RACE cDNA Amplification Kit (Clontech Laboratories, Inc., Mountain View, CA, USA). To find the supposed nucleic acid sequence, a local BLAST was conducted using the program of blastn in bioedit sequence alignment editor and the nucleotide sequence of *AmBMT* (GenBank accession No: AY443006) was used as a template for homologous alignment (Altschul et al., 1997; Hehmann et al., 2004). According to the alignment results, the highly conserved region of the sequence labeled as *comp34798_c0_seq1* was used as a template to design gene-specific primers (BMT-GSP2, BMT-GSP1, BMT-NGSP2, and BMT-NGSP1, Supplementary Table S1) for amplification of 5′- and 3′-end fragments by SMARTer^TM^ RACE cDNA Amplification Kit. The 5′- and 3′- end fragments were sequenced to assemble full length cDNA, which was then deposited to the National Center for Biotechnology Information (NCBI) with the accession number of KU359196.

### Protein Expression and Purification

To analyze the activity of BMT, the open reading frame (ORF) was amplified using primers 28a-*SacI*-F (5′-GAGCTCATGG CAGGAATGAAGACT-3′) and 28a-*NotI*-R (5′-GCGGCCGC CTACTTGGAAAATTCCATAAT-3′) with additional enzyme recognition sites *SacI* and *NotI* (underlined), respectively (Supplementary Table S1). Then, the amplified fragment was restricted with *Sac I* and *Not I* and ligated into pET28a plasmid with the same digested cloning site to generate pET28a-BMT. Subsequently, the recombinant plasmid pET28a-BMT was transformed into *E. coli* BL21 (DE3) for heterologous expression in a selective medium (LB, Luria–Bertani, with 50 mg/L kanamycin or 20 g/L agar whenever necessary). For details, individual colony was picked into a 25 ml LB medium for overnight culture. Then, 1% of the cultures was inoculated in the same medium and grown to an OD_600_ of 0.6–0.8, at which the cultures were induced by addition of 1 mM isopropyl β-D-1-thiogalactopyranoside (IPTG). After growing about 16 h at 16°C, the cultures were harvested and used for protein purification or BMT activity analysis. For *in vivo* enzyme activity analysis, 100 μM bergaptol was added to the cultures for an additional 24 h at 37°C. After centrifugation, the supernatant was used to measure the bergapten yield by high performance liquid chromatography (HPLC). For protein purification, ammonium sulfate precipitation was conducted according to method described by Lo et al. (2012) since Ni-nitrilotriacetic acid (NTA) column is not suitable for BMT purification (Hehmann et al., 2004; Lo et al., 2012). For sodium dodecyl sulfate-polyacrylamide gel electrophoresis (SDS-PAGE) analysis, the precipitation was mixed with 2x sample buffer [2% (w/v) SDS and 4% (v/v) 2-mercaptoethanol] and fractionated by 15% (w/v) SDS-PAGE. After electrophoresis, the gel was stained with 0.25% coomassie blue R-250, 10% glacial acetic acid, and 45% methanol for 6 h, and destained with 10% glacial acetic acid, and 10% methanol for about 6 h.

### BMT Activity and HPLC/Electrospray-Ionization Quadrupole Time-of-Flight Mass Spectrometry (Q-TOF MS) Analysis

After the BMT protein was purified, different reactions were conducted according to the previous reports with some minor modifications (Hehmann et al., 2004; Lo et al., 2012). For details, reaction was started with addition of 5 mM SAM in a total volume of 200 μl containing 200 mM potassium phosphate buffer (PBS, pH 7.5), some 10 μg protein and 1 mM bergaptol. The reaction was conducted in a temperature control instrument with a constant temperature of 25°C for 60 min and ended with addition of 20 μl 20% trichloroacetic acid. For HPLC analysis, the reaction broth was extracted with ethyl acetate (threefold of reaction broth) for three times and the combination of ethyl acetate was evaporated to dryness using a stream of nitrogen gas. The residue of the extract was suspended in 1 ml of methanol for further analysis. HPLC equipped with a reversed phase C18 column (XDB-C18, 5 μm; Agilent, USA) was conducted to analyze the concentration of bergapten at a flow of 1 ml/min. The solvent gradient conditions A (H_2_O): B (methanol; v/v) was as follows: 0 min, 90:10; 30 min, 10:90; 31 min, 90:10; 36 min, 90:10. This HPLC system was connected to Q-TOF MS spectrometer (Agilent Technologies, Santa Clara, CA, USA) equipped with an electrospray interface to identify the products. The conditions of the ESI source were as follows: drying gas (N_2_) flow rate, 8.0 l/min; drying gas temperature, 300°C; nebulizer, 241 kPa (35 psig); capillary voltage, 4000 V; fragmentor voltage, 150 V; collision energy, 30 eV; skimmer voltage, 60 V, and octopole radio frequency, 250 V.

### Expression and Quantitative Real-Time PCR (qPCR) Analysis

To determine the expression level of BMT in different tissues (roots, stems, and leaves), qPCR analysis was performed using the SYBR Green PCR Master Mix (Vazyme, Nangjing, China) with LightCycler 480 instrument (Roche Molecular Biochemicals, Mannheim, Germany). The reaction was conducted in a mixture containing 10 μl 2x SYBR Green Master Mix Reagent, 10 ng cDNA and 4 μM gene-specific primers. The cycling conditions were as follows: 1 cycle of 95°C for 5 min, 40 cycles of 95°C for 10 s, and then 60°C for 30 s, followed by 1cycle of 95°C for 15 s, 60°C for 60 s, and 95°C for 15 s. The mean value of three replicates was normalized with *SAND family protein* (*SAND*) according to the report before (Zhao et al., 2016). PCR amplification was performed using specific primers listed in Supplementary Table S1. To eliminate the influence of the possible exited close homologous of *PpBMT*, two gene-specific primers were used. The relative expression levels were calculated by 2^-ΔΔCT^ method (Pfaﬄ, 2001).

### Subcellular Localization Analysis

The specific primers 5′-CCATGGCAGGAATGAAGACTAGTC C-3′ (1302-*NcoI*-F) and 5′-AGATCTACCATCTTGGAAAATT CCATAATCC-3′ (1302-*BglII*-R) containing the *NcoI* and *BglII* sites (underlined), respectively, were used to amplify the cDNA fragment encoding the full-length BMT protein (Supplementary Table S1). Then, The PCR product was inserted into pCAMBIA-1302 at the 5′-terminal of the green fluorescence protein (GFP) gene under the control of the cauliflower mosaic virus (CaMV) 35S promoter to construct pCAMBIA-1302-BMT plasmid. Subsequently, the constructed pCAMBIA-1302-BMT plasmid and pCAMBIA-1302 empty vector were transformed into *Arabidopsis* protoplasts, respectively, using polyethylene glycol (PEG)-mediated transient gene expression (Wu et al., 2009). The untransformed *Arabidopsis* protoplasts were used as control. After 16 h transformation, protoplasts were observed with a laser scanning confocal microscope (LSM 780; Carl Zeiss, Jena, Germany) using 20×/0.8 Plan-Apochromat, 40×/1.2 W C-Apochromat or 63×/1.4 Oil Plan-Apochromat in multitrack channel mode.

### Bioinformatics Analysis, Homology Modeling, and Site-Directed Mutagenesis

**FIGURE 2:**
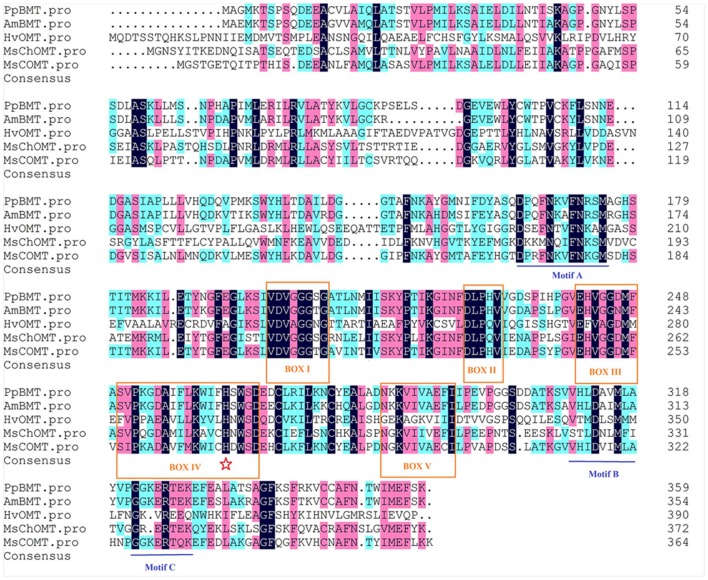
**Sequence alignment of *PpBMT* with other plant OMTs.** Boxed amino acids (regions I–V) represent conserved regions. Residues involved in SAM binding, substrate binding, catalytic center are indicated by boxes, lines and pentagram, respectively. Accession numbers: *AmBMT* (AY443006), *HvOMT* (CAA54616), *MsChOMT* (AAB48059), *MsCOMT* (AAB46623).

**FIGURE 3:**
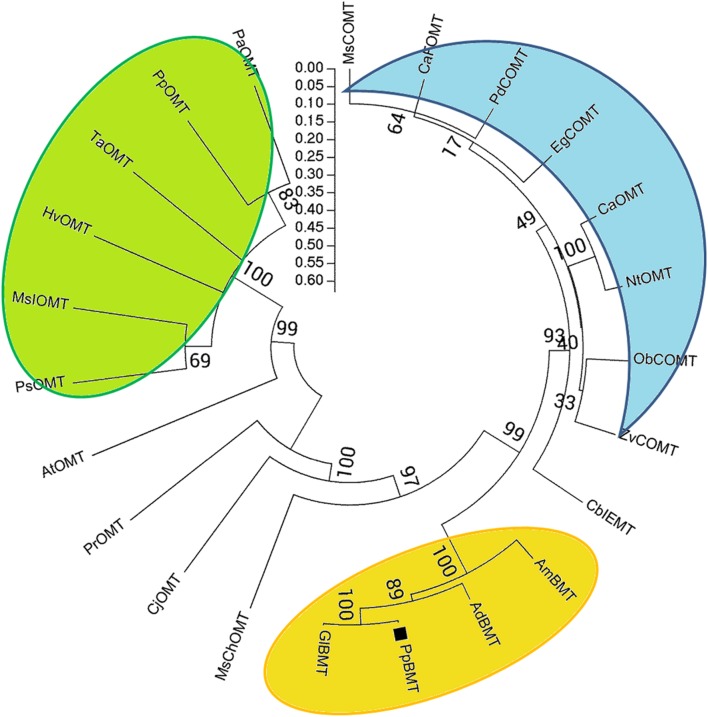
**Phylogenetic relationships between *PpBMT* and other OMTs.** Neighbor-joining phylogram for amino acid sequences of PpBMT and other OMTs were drawn by ClustalW, showing the result of 1000 bootstrap tests using MEGA5 software. Accession numbers: *AmBMT* (*Ammi majus* AY443006), *GlBMT* (*Glehnia littoralis* AB363638), *AdBMT* (*Angelica dahurica* AEO21927.1), *MsChOMT* (*M. sativa* AAB48059), *MsCOMT* (*M. sativa* AAB46623), *HvOMT* (*Hordeum vulgare* CAA54616), *AtOMT* (*Arabidopsis* NP_195242.1), *CaOMT* (*Capsicum annuum* Q9FQY8.2), *NtOMT* (*Nicotiana tabacum* CAA52461.1), *CaFOMT* (*Chrysosplenium americanum* Q42653.1), *CbIEMT* (*Clarkia breweri* O04385.2), *EgCOMT* (*Eucalyptus gunnii* P46484.1), *CjOMT* (*Coptis japonica* Q39522.1), *PsOMT* (*Pisum sativum* O24305.1), *PdCOMT* (*Prunus dulcis* Q43609.1), *PpOMT* (*Pyrus pyrifolia* BAA86059.1), *MsIOMT* (*M. sativa* O22309.1), *ObCOMT* (*Ocimum basilicum* Q9XGW0.1), *PrOMT* (*Pinus radiata* AAD24001.1), *PaOMT* (*Prunus armeniaca* AAB71213.1), *TaOMT* (*Triticum aestivum* AAD10485.1), *ZvCOMT* (*Zinnia violacea* Q43239.1).

The theoretical isoelectric point (pI) and molecular weight (Mw) were predicted by online compute pI/Mw tool^1^. Multiple sequence alignment was performed using DNAMAN (Lynnon Corp., Pointe-Claire, QC, Canada), and the five sequences of OMT are displayed in **Figure 2**. Neighbor-joining phylogram for phylogenetic analysis of amino acid sequences of *PpBMT* and other OMTs were drawn by ClustalW, showing the result of 1000 bootstrap tests using MEGA5 software (Tamura et al., 2011). The protein sequences and accession numbers of OMT displayed in **Figure 3** are listed in the legend of **Figure 3** or Supplementary Table S2 according to the previously report (Zubieta et al., 2001, 2002; Louie et al., 2010; Lo et al., 2012; Green et al., 2014). Homology modeling was conducted according to the method described by Larbat et al. (2007) and use *MsCOMT* as the template (PDB accession number: 1KYZ; Zubieta et al., 2002). Then, molecular docking was proceeded with SAM and bergaptol to generate the complexes of *PpBMT*-SAM and *PpBMT*-bergaptol, respectively. According to the results, the site-directed mutagenesis experiment was conducted using overlap PCR method and the primers used in this section were listed in Supplementary Table S1. The products of site-directed mutagenesis were then transferred into *E. coli* to analyze bergapten yield.

### Statistical Analysis and the Preparation of Graphs

Three biological and technical replicates were used to obtain the data. Unless the special comments, the data were presented as mean of triplicate experiments ±SD. Unless produced with the specific software (DNAMAN, for instance), the original graphs were generated using OriginPro 8 (OriginLab Corporation, Northampton, MA, USA) or Microsoft Office PowerPoint 2010. The graphs were merged with Adobe PhotoShop CS6 whenever necessary.

## Results

### Cloning and Bioinformatics Analysis of *PpBMT* cDNA from *P. praeruptorum*

A blastn program was conducted according to the BMT sequences reported previously and our transcriptome database of *P. praeruptorum* (Hehmann et al., 2004; Ishikawa et al., 2009; Lo et al., 2012; Zhao et al., 2015). According to the *E*-value of alignments, only one unigene named as *comp34798_c0_seq1* was selected as a candidate BMT gene for further investigation (*E*-value = 0, data no given). Then, the full length cDNA was obtained by RACE with gene-specific primers designed according to the nucleotide sequence of *comp34798_c0_seq1.* The obtained full-length cDNA (GenBank accession No: KU359196) comprised a 1080-bp ORF, 13- and 263-bp 5′ and 3′ untranslated regions and 29-bp poly A tail (Supplementary Table S3). The ORF encodes a 359-residue protein (**Figure 2**, *PpBMT*) with a predicted molecular weight of ~39 kDa and a pI of 5.6. The deduced amino acid sequence was compared with other reported OMTs using DNAMAN (**Figure 2**). The deduced amino acid sequence showed a low similarity with other reported OMTs from *H. vulgare* (29% identity) and *M. sativa ChOMT* (44.9% identity). However, it shares a high similarity with OMT from *A. majus* (87% identity). In addition, the five conserved regions (in boxes) and speculative residues involved in catalytic center (asterisk), SAM (boxes), and substrate binding (line) are also displayed either in boxes or different marks (**Figure 2**) (Zubieta et al., 2001). For details, the five conserved regions might be involved in SAM binding while motif A, B, and C are participated in bergaptol binding. The phylogenic analysis of OMT sequences (**Figure 3**) indicated that these enzymes are organized in clusters and different OMTs with a specific enzymatic function are clustered into the same clusters (indicated with different colors). To be specific, the cloned PpBMT sequence belongs to the same cluster as the *AmBMT* (orange) and shares the highest sequence identify to *Glehnia littoralis bergaptol-O-methyltransferase* (*GlBMT*, AB363638). The high sequence similarity revealed that they might display a same enzymatic function such as substrate specificity, optimal pH, reaction temperature and catalytic mechanism.

### Heterologous Expression, Activity Analysis, and Substrate Specificity of BMT

**FIGURE 4:**
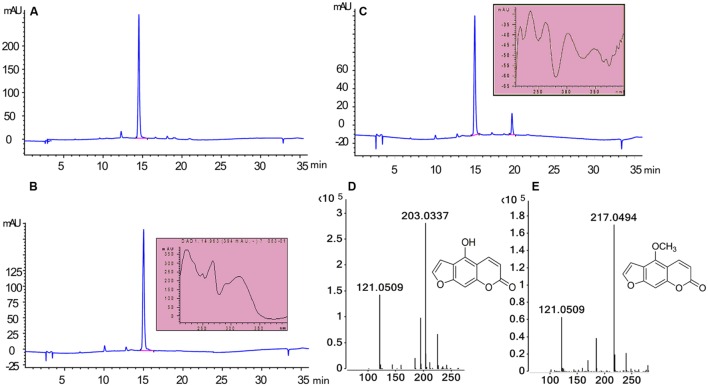
**Enzymatic characteristics of PpBMT expressed in *E. coli.* (A)** *E. coli* without transform. **(B)** *E. coli* containing empty vector pET28a. **(C)** *E. coli* containing recombinant plasmid pET28a-BMT. **(D)** MS fragmentation of substrate. **(E)** MS fragmentation of products. Ultraviolet absorption spectra and the structure of substrate and product are also shown beside the corresponding peak.

**FIGURE 5:**
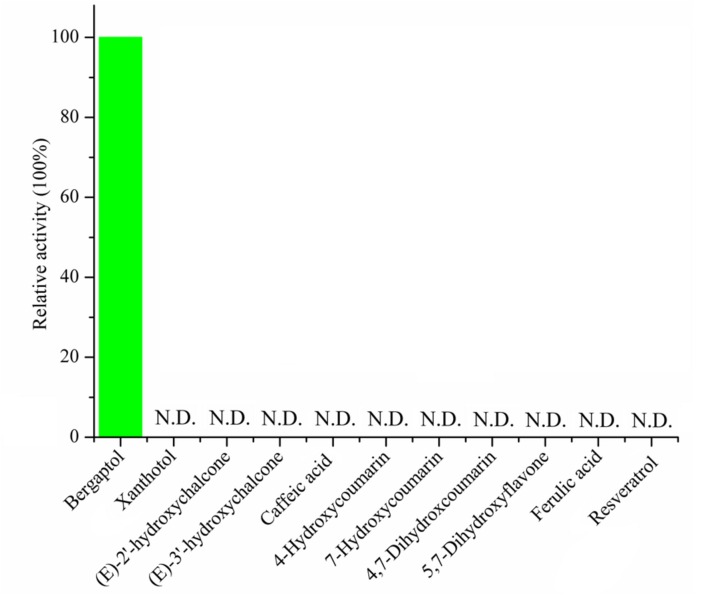
**Substrate specificity of recombinant *PpBMT.*** The activity of the enzyme toward bergaptol is set as 100% and the structure of candidate substrates are drawn in the picture. N.D. represents no detectable.

To assess the activity of the BMT, the ORF was cloned with 5′-SacI and 3′-NotI enzyme recognition sites and then transferred into *E. coli* BL21 (DE3) through the expression vector pET28a. After inducing with IPTG at different temperatures (ranging from 11°C to 42°C), SDS-PAGE analysis was employed and the results indicated the recombinant bacteria contained an overexpressed protein with a molecular mass of approximately 39 kDa, which was consistent with the predicted molecular weight (Supplementary Figure S1). In addition, the results also indicated that reducing the temperature could somehow improve the protein amount of recombinant enzyme. Hence, an inducing temperature of 16°C was selected for the coming experiments (Supplementary Figure S1). To analyze the activity of BMT, some 100 μM bergaptol was added into the medium after inducing for another 24 h at 37°C. The supernatant was used for activity analysis by HPLC and the results were listed in **Figure 4**. As it was indicated, the strain containing the recombinant plasmid (**Figure 4C**) pET28a-BMT displayed a new peak against that harboring empty vector (**Figure 4B**) or strain without plasmids (**Figure 4A**). To further confirm the correctness of the products, Q-TOF MS spectrometer was employed and the [M+H] signal of 217 (**Figure 4E**) indicated bergaptol had been transferred into bergapten (**Figure 4D**) by *PpBMT*. To investigate whether PpBMT had the same catalytic activity *in vitro* as observed *in vivo*, the protein was purified after a 16 h period of inducing at 16°C (Ishikawa et al., 2009; Lo et al., 2012). Then, the *in vitro* functional activity of *PpBMT* was conducted with the purified protein in a volume of 200 μl in 96-well plate with addition of SAM, bergaptol and PBS (pH 7.5). And, the same result was observed *in vitro* as observed *in vitro*. To investigate the substrate specificity, different substrates listed in Supplementary Figure S2 were added into the reaction broth in place of bergaptol, respectively. The reaction broth was harvested for analysis and the results showed that no object products were produced (**Figure 5**). The results indicated that *PpBMT* had narrow substrate specificity toward bergaptol. For instance, xanthotol, an isomer of bergaptol, could not be converted by *PpBMT* although it only has a minute difference with bergaptol in the position of hydroxyl.

### Expression Profile in *P. praeruptorum*

**FIGURE 6:**
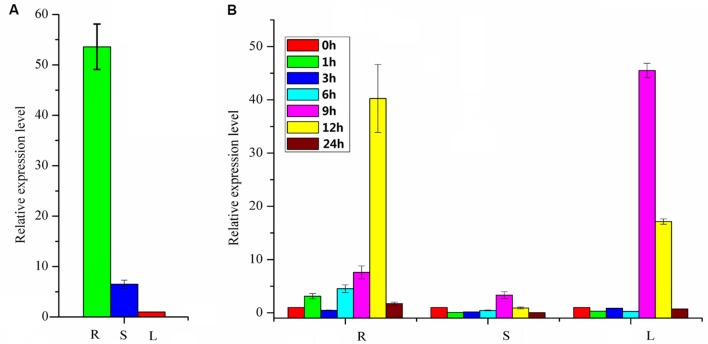
**Expression profiles of *PpBMT.* (A)** Tissue-specific expression. **(B)** Gene expression after MeJA treatment. R, roots; S, stems; L, leaves. 0, 1, 3, 6, 9, 12, and 24 h represent the time interval after MeJA treatment. The expression level of leaves in **(A)** and 0 h in **(B)** were set as reference in each group. Each bar represents the mean value results from the mean of triplicate experiments ±SD.

The expression profile of the PpBMT in various tissues of *P. praeruptorum* was investigated using qRT-PCR with the total RNA from roots, stems, and leaves. As a result, the expression level of roots tends to be higher than stems and leaves. Especially, the expression level in roots was ten folds higher than in stems and 54-fold higher than in leaves (**Figure 6A**). The expression mode was consistent with CYP98A22, another enzyme involved in coumarins biosynthesis (Karamat et al., 2012). When it is subjected to methyl jasmonate (MeJA) treatment, different expression profile was observed and the results showed that MeJA can significantly improve the expression level of *PpBMT*. In addition, time-course changes of MeJA induced gene expression were also investigated and the results indicated that the expression level can be largely enhanced in root at 12 h after MeJA addition. However, this tendency was observed at 9 h in leaves and there is no apparent difference in stems (**Figure 6B**). The results indicated that different tissues showed a different response to MeJA. Owing to the fact that the *P. praeruptorum* genome is not sequenced, it is highly possible that a close homology of *PpBMT* exist. To eliminate the influence of the possible exited close homology of *PpBMT*, another gene-specific primer was used to investigate the expression profile of *PpBMT* in *P. praeruptorum* and the results were listed in Supplementary Figure S3. As the result, a similar expression profile was observed which indicated that that the qPCR analysis method used in this study was credible.

### Subcellular Localization of *PpBMT*

**FIGURE 7:**
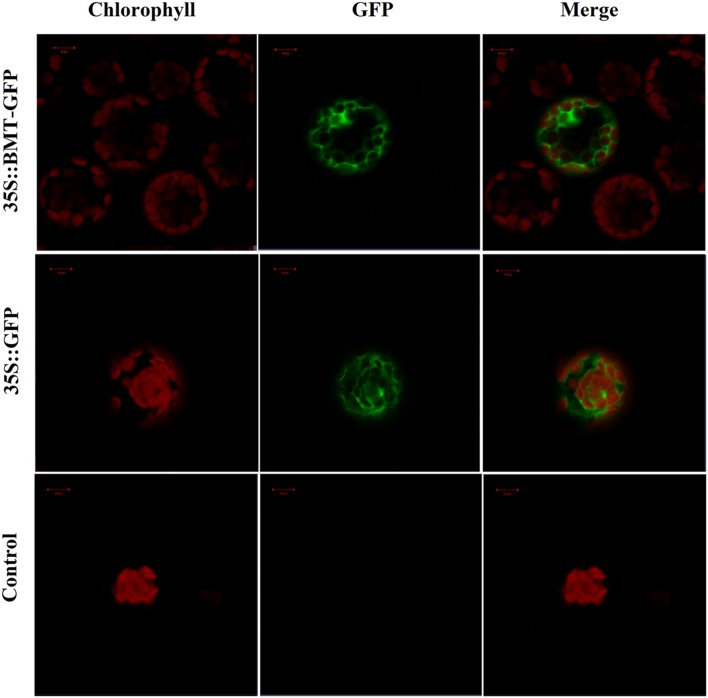
**Confocal fluorescence microscopy images of *PpBMT*-GFP fusion protein in transgenic *Arabidopsis* protoplasts.** Control, *Arabidopsis* protoplasts without be transformed any vectors. 35S::GFP, *Arabidopsis* protoplasts contains empty vector. 35S::*PpBMT*-GFP, *Arabidopsis* protoplasts contains constructed pCAMBIA-1302-BMT plasmid. The photographs were taken in the red channel (**Left**, chlorophyll), in the blue channel (**Middle**, GFP) and in their combination (**Right**, merge). Auto-fluorescence of chlorophylls in chloroplast was used as a control for plastid targeting. The images were obtained at 16 h of transformation. Scale bars show 10 μm.

In order to investigate the subcellular localization of *PpBMT*, the ORF of *PpBMT* was inserted into pCAMBIA1302 at the 5′-terminal of the GFP gene under the control of the CaMV 35S promoter. Then, a PEG mediated transient gene method was employed to transfer the constructed pCAMBIA-1302-BMT plasmid into protoplast of *Arabidopsis* according to the previous report (Wu et al., 2009). Owing to the fact that the stop codon of BMT had been deleted, the *PpBMT* protein could be fused with GFP at the N terminus (BMT-GFP). Hence, the GFP can be corporately expressed with *PpBMT*. At the same time, the empty vector and the untransformed *Arabidopsis* protoplasts were used as control. The subcellular localization results were analyzed by confocal laser scanning microscopy and the results were depicted in **Figure 7**. For *Arabidopsis* protoplasts containing BMT-GFP, a green fluorescence was observed full of the cell and could not be merged with the auto-fluorescence of chlorophylls, which suggests that the fusion protein is located in cytoplasm. By contrast, the protoplast transformed with the empty GFP vector alone could not be located and green fluorescent signals in the cytoplasm either. In addition, non-transformed protoplasts (control) for auto-fluorescence with the same acquisition parameters were also shown and no green signal was observed.

### Catalytic Mechanism

**FIGURE 8:**
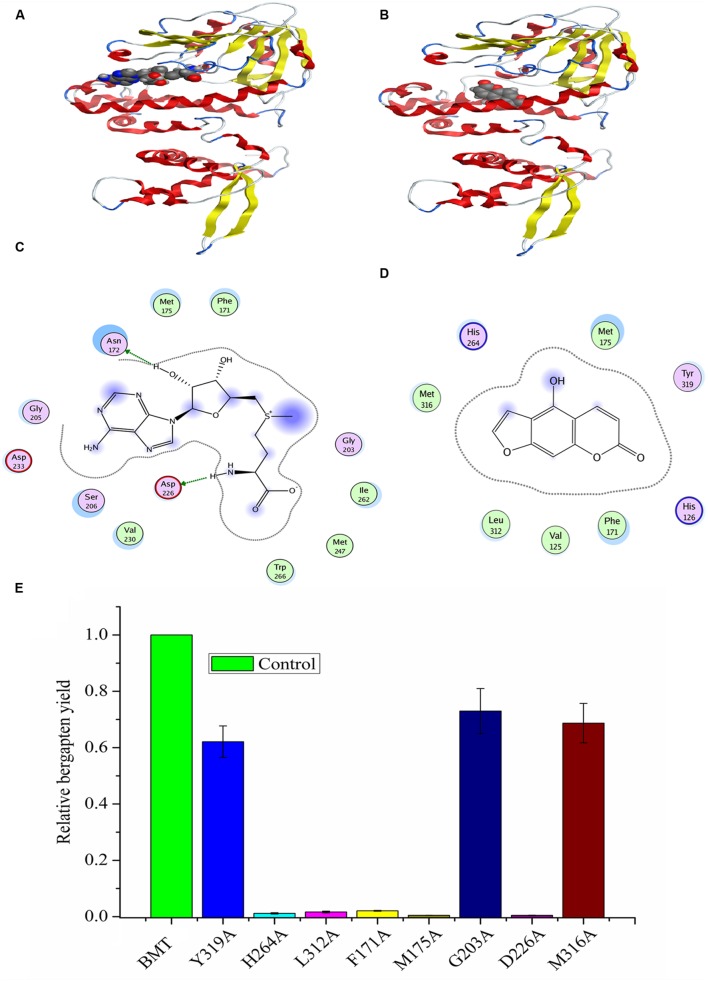
**Homology modeling and docking of *PpBMT* with SAM and bergaptol. (A)** Three-dimensional model of PpBMT-SAM. **(B)** Three-dimensional model of *PpBMT*-bergaptol. **(C)** Two-dimensional model of PpBMT-SAM. **(D)** Two-dimensional model of *PpBMT*-bergaptol. **(E)** Site-directed mutagenesis of *PpBMT* according to the docking results. Protein was depicted in line and the substrate was shown in spheres. The activity of *PpBMT* was set as reference. Each bar represents the mean value results from the mean of triplicate experiments ±SD.

Since there are no crystal structures of BMT, at the beginning of the experiment, an online blast was conducted in SWISS-MODEL server to find the template for homology modeling. As a result, a protein named as *caffeic acid/5-Hydroxyferulic acid 3/5-O-Methyltransferase* (PDB accession No: 1KYZ) was chosen as the template for the high similarity (Zubieta et al., 2002). Then, a MOE program (Chemical Computing Group, Montreal, QC, Canada) was employed to construct the homology model according to the method described by Larbat et al. (2007) and the optimal model was listed in Supplementary Figure S4A. Finally, the model structure was minimized using the CHARMm22 force field to an energy gradient of <0.01 kcal/mol Å. The docking simulation was conducted according to the constructed model using SAM and bergaptol as substrates, then, the confidence level of docking result was confirmed by Ramachandran plot (Supplementary Figure S4). The protein/substrate complex was minimized using MMFF94 force field and the three dimensional (3D) structures are listed in **Figures 8A,B**. As is depicted, the substrates, SAM and bergaptol are all inclusived by the *PpBMT* binding pocket. In an ongoing effort to be more visible, a 2D docking model was also included in **Figures 8C,D**. The structure modeling revealed that the amino acid residues constituting the substrate-binding pocket were determined to be Y319, L312, F171, M316, G203, M175, D226, H264, etc. Those amino acid residues may interact with either SAM or bergaptol. For instance, D226 may be involved in SAM/SAH binding and F171 and/or M175 might participate in substrate binding. Additionally, H264 might play an important role in the catalytic center. To confirm those hypotheses, site-directed mutagenesis was conducted according to the results reported in docking. As is shown in **Figure 8E**, the mutants F171A, M175A, D226, H264A, and L312A nearly totally lost the BMT activity. Hence, these residues may play a critical role in substrate binding or catalytic. However, details need to be discussed according to the results in **Figure 2** and previous reports (Zubieta et al., 2001, 2002; Louie et al., 2010; Green et al., 2014).

## Discussion

Coumarins are important components of phenylpropanoids which have diverse biological activities and great pharmacological importance to human being. Despite plenty of papers focused on their chemical structures and biological activities (Ling-Yi et al., 1996; Yang et al., 1999; Yu et al., 2012), little is known regarding the biosynthesis and transport mechanism of coumarins in plants (Zhao et al., 2015). As an important reaction type, methylation by SAM dependent OMTs is a common modification in natural product biosynthesis (Lo et al., 2012). However, the coumarins biosynthesis related publications are limited and no additional reactions have been reported except the conversion from bergaptol to bergapten (**Figure 1**). As the main source of coumarins in China Pharmacopeia (Shao, 2010), *P. praeruptorum* plays an important role in the treatment of anemopyretic cold, cough with abundant phlegm and congested chest (Zhou et al., 2014). However, no enzyme involved in the reaction of methoxylation and other reactions has been cloned from *P. praeruptorum* in the past years.

Previously, there were reports with focus on cloning and constitutive expression of BMT in *E. coli* or in *Glehnia littoralis* cell cultures (Hehmann et al., 2004; Ishikawa et al., 2009; Lo et al., 2012). And, the effects of pH, temperature, and metal ions on the recombinant BMT activity, substrate specificity and enzyme kinetics were investigated in details. For instance, a pH of 7–8 and a temperature of 30–40°C were suitable for BMT activity and addition of Cu^2+^ and Ni^2+^ could significant inhibit the BMT activity (Hehmann et al., 2004; Ishikawa et al., 2009; Lo et al., 2012). However, little is known regarding its plant physiology characteristics such as tissue expression profile and subcellular localization. And there are also no systematic study regarding its catalytic mechanism. Hence, in this study, a detail investigation on the catalytic mechanism and plant physiology characteristics of BMT were put forward apart from the basic biochemical characterization of the enzyme. And the systemic study summarized here will enlarge our knowledge on other OMTs and provide more information in investigating the biosynthesis mechanism of coumarins in *P. praeruptorum.*

At the beginning of the article, the biosynthesis pathway of coumarins in *P. praeruptorum* involved in methylation was described according to the previous reports (Hauffe et al., 1986; Bourgaud et al., 2006; Zhao et al., 2015). As is depicted, methylation participated in the formation of structure modified, namely bergapten, xanthotoxin, isopimpinellin, and so on. However, only the gene encoding BMT was cloned and functionally characterized, despite the reports of other enzymes acting on xanthotoxin or isopimpinellin (Innocenti et al., 1983; Hauffe et al., 1986). The description summarized in **Figure 1** indicated that mountains of work need to be done to investigate the biosynthesis mechanism of coumarins. Amino acids sequence alignment indicated that, similar to other OMTs, *PpBMT* had five high conversed regions and three motifs might serve as substrate binding area, catalytic center and/or hydroxylated substrate binding area (**Figure 2**). On the other hand, the high similarity to AmBMT indicated that they may have the same enzyme properties or kinetic parameters. The hypothesis can also be proved in phylogenetic analysis (**Figure 3**) which also indicated the high similarity between the BMTs from *A. majus, A. dahurica*, *G. littoralis, and P. praeruptorum.* It has also been reported that the activity profile of the recombinant *AmBMT* was fully compatible with the BMT extracted from *A. majus* cells, and the pH dependency was corresponded to that of the BMTs from *P. crispum* or *R. graveolens* (Hehmann et al., 2004). In addition, the effects of metal ions and the kinetic parameters have high similarity between *GlBMT*, *AmBMT*, and *AdBMT* (Hehmann et al., 2004; Ishikawa et al., 2009; Lo et al., 2012). Hence, the enzyme properties and the kinetic parameters are not included in this study.

As an important composition of BMT activity study, the expression profiles play a significant role in plant physiology and compound synthesis. The different space-time expression mode was considered as a self-regulation to the surrounding environment or inner steady by synthesis of related compounds (Gutierrez et al., 1995; Chong et al., 2002). Hence, a tissue-specific expression mode was investigated and the results indicated that the *PpBMT* had a high expression level in roots (**Figure 6A**). There is also a report showing that higher bergapten content was detected in the roots of *P. praeruptorum* (Zhao et al., 2015). In hypothesis, the coumarins are initial composited by cytochrome P450s and then transferred to roots or certain parts of plant or organelles for structure modifications (Zhao et al., 2015). Thus, it is reasonable that BMT can be highly expressed in roots since it participates in the formation of modified compound by methylation. It is consistent with the fact that we usually choose the roots of *P. praeruptorum* as medicinal parts in clinical trials (Shao, 2010). There are also reports in studying the tissue-specific distribution of furanocoumarins in *Apiaceae* and *Rutaceae* despite the reason for this phenomenon which remained unresolved in those years (Nitao and Zangerl, 1987; Milesi et al., 2001). In addition, a MeJA induced gene expression experiment was conducted to investigate the gene expression behavior toward exogenous stimulus in roots, stems, and leaves, respectively. Obviously, BMT displayed a strong response to MeJA and an immediately enhanced expression was observed at 9 h after MeJA treatment. The up-down expression mode was consistent with the other genes involved in coumarins biosynthesis (Zhao et al., 2015).

Mountains of studies have focused on the role of coumarins as key allelochemicals, but the physiological relevance of coumarins reaches far beyond in the producing plants (Bourgaud et al., 2006). The potential role of simple coumarins as hormones and signaling molecules has been widely reported in plant kingdom than previously assumed in the past decade (Yang et al., 1999; Ndong et al., 2003; Bednarek et al., 2005). However, details on functional insight will remain unsolved until the mechanism and regulation of coumarin biosynthesis and subcellular localization are clearly interpreted. Furthermore, plenty of enzymes are involved in coumarins biosynthesis and different enzymes tend to have a different subcellular localization (Zhao et al., 2015). Hence, interpretation of the subcellular localization of coumarins biosynthesis related enzyme will be a key work in biosynthesis pathway study. In this study, the subcellular localization of BMT was reported for the first time and the results indicated that it was located in cytoplasm (**Figure 7**). The results will give us useful information in coumarins biosynthesis study. And thus, the tissue, organ and subcellular-specific biosynthesis and transport mechanism could be ultimately illuminated (Bourgaud et al., 2006; Zhao et al., 2015).

Another question to be interpreted is the catalytic mechanism of *PpBMT*. Despite a mass of paper has been published in the past 14 years regarding the crystal structure of OMT (Zubieta et al., 2001, 2002; Louie et al., 2010; Pan et al., 2014), no reports specialized in the catalytic mechanism of BMT. Thus, in this study, a structure based computational designing method was combined to investigate the catalytic mechanism of BMT. As is indicated, the mutants F171A, M175A, D226A, H264A, and L312A somewhat totally lose the BMT activity (**Figure 8**). This phenomenon is consistent with the previous report that one of them may be involved in catalytic center, substrate binding or the correct orientation (Zubieta et al., 2001, 2002; Louie et al., 2010; Pan et al., 2014). For example, His-264 is a residue reported to be involved in catalytic center (Zubieta et al., 2002) and it is also highly conserved as shown in **Figure 2**. For details, the hydroxyl group of bergaptol is deprotonated by His-264, facilitating the transfer of the reactive methyl group of SAM to the newly formed phenolate anion (Zubieta et al., 2002). The catalytic mechanism appears to be favorable for S_N_2-nucleophilic attack by the activated hydroxyl group of bergaptol on the reactive methyl group of SAM (Zubieta et al., 2001; Louie et al., 2010). F171, M175, and L312 are residues in motif A and B in **Figure 2** and they are reported to be involved in binding of hydroxylated substrate. Hence, mutation of them can also lose the enzyme activity (Zubieta et al., 2001, 2002; Pan et al., 2014). In addition, a nearest observed distance appeared in **Figure 8D** may also indicate that F171, M175, and L312 are important to bergaptol–*PpBMT* interaction. Another important residue, D226, also a highly conserved residue in **Figure 2** (Box I) is proved to be involved in SAM-binding and mutation of D226 can also affect the activity of *PpBMT* (Zubieta et al., 2001, 2002). **Figure 8C** also indicated that Asp-226 could have a strong hydrogen bond with the hydrogen of amino in SAM. Although the activity of mutants G203A, M316A, and Y319A still remains, a large extent of activity decline was observed, indicating that these residues may participate in right location of substance or the correct orientation of the key activity residues (Zubieta et al., 2002; Pan et al., 2014). For instance, these functions of BMT in *P. praeruptorum* might be mostly like the one in COMT from alfalfa, in which Glu-329, Glu-297, and Asp-270 are adjacent to the His and contribute to the orientation of His-269 (Zubieta et al., 2002). There are also report indicated that M316 (M317 in *L. perenne LpOMT1*) may also be involved in substrate binding (Louie et al., 2010) and the G203 consists of a Gly-rich segment (**Figure 2**) forms one wall of a channel that is occupied by the ribose ring and the aliphatic portion of the methionine/homocysteine (Met/Hcy) moiety (Louie et al., 2010). Although a main catalytic mechanism has been stated in this study, the special function of other residues were not fully understood despite in a simulation experiment (**Figures 8C,D**), especially the highly conserved regions displayed in **Figure 2**. Additionally, little is known about the strict substrate specificity (**Figure 5**) although computer aided site-directed mutagenesis method was conducted to investigate the possible mechanisms of transferring methyl from SAM to bergaptol by BMT. Despite the high sequence similarity and the same conserved domains, distinctions of different BMT are also existed from kinetic parameters to substrate selectivity. Briefly, more work needs to be conducted to explain the reasons for these distinctions.

## Conclusion

The paper reported here, for the first time, describes the cloning and biochemical characterization of a BMT cDNA from *P. praeruptorum*. The protein sequence alignment indicated that some local regions are highly conserved but the phylogenetic tree analysis displays it has a big distinction to other OMTs. The results indicating that it might have special kinetic parameters and substrate selectivity despite the similar functions to other OMTs. This phenomenon is also confirmed by substrate specificity experiment, in which a narrow selectivity was observed. The gene of *PpBMT* is highly expressed in roots, which is in accordance with the reported bergapten accumulation behavior in *P. praeruptorum*. Subcellular localization indicates that the enzyme is located in cytoplasm *in planta*. In addition, the computer aided site-directed mutagenesis experiment indicates that mutation of residue H264 affects BMT catalysis, whereas substitutions at residues F171, M175, D226, and L312 affect substrate binding. The investigation has added to the knowledge of key genes in coumarins biosynthesis in *P. praeruptorum*, which will also enlarge the functional insight into the mechanism and regulation of biosynthesis pathway in other plants.

## Author Contributions

YZ and NW conceived and designed the experiments. ZZ and SX analyzed the data and write the manuscript. CH and WW performed the bioinformatics experiment. YZ, NW, and TL performed the subcellular localization and tissue expression. JL and TL participated in the design of the study. YC helped to analyze the data and draft the manuscript. LK, JL, TL, and ZZ conducted in compound identification. All authors read and approved the final manuscript.

## Conflict of Interest Statement

The authors declare that the research was conducted in the absence of any commercial or financial relationships that could be construed as a potential conflict of interest.

## Footnotes

1 http://web.expasy.org/compute_pi/

## Supplementary Material

FIGURE S1 **SDS-PAGE analysis of protein expression**. DE3 indicated *E. coli* BL21 (DE3). PET28a indicated the strain containing empty vector. Then, 11–41 indicated that the strain (contains pET28a-*PpBMT*) was induced at 11–41°C. Markers are displayed in the left and the protein molecular weight of 97.2, 66.4, 44.3, 29, 20.1, and 14.3 kDa are listed from top to the bottom. The arrow was used to point the expressed protein in the figure.

FIGURE S2 **The different substrates used in this study**.

FIGURE S3 **Expression profiles of *PpBMT* using another gene-specific primer. (A)** Tissue-specific expression. **(B)** Gene expression after MeJA treatment. R, roots; S, stems; L, leaves. 0, 1, 3, 6, 9, 12, and 24 h represent the time interval after MeJA treatment. The expression level of leaves in **(A)** and 0 h in **(B)** were set as reference in each group. Each bar represents the mean value results from the mean of triplicate experiments ±SD.

FIGURE S4 **Homology model and docking of *PpBMT.* (A)** The best homology model of *PpBMT*. **(B)** One of the Ramachandran plots of docking.
