# Supplementary figures and images for: Cloning, Functional Characterization, and Catalytic Mechanism of a Bergaptol O-Methyltransferase from Peucedanum praeruptorum Dunn
Source: Front Plant Sci. 2016 May 25;7:722. doi: 10.3389/fpls.2016.00722 (PMC4879325; doi:10.3389/fpls.2016.00722)

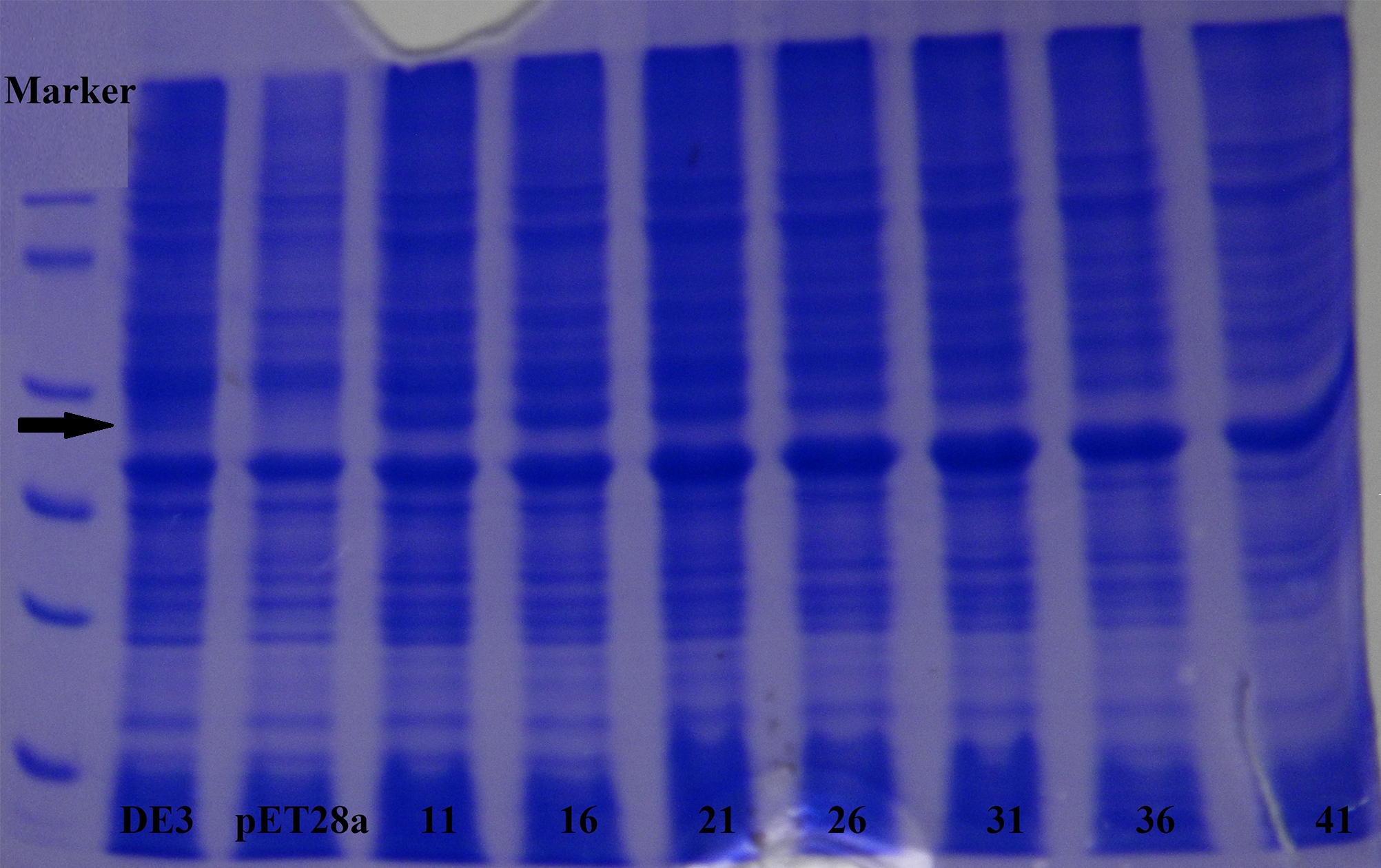

Supplement: FIGURE S1 — SDS-PAGE analysis of protein expression. DE3 indicated E. coli BL21 (DE3). PET28a indicated the strain containing empty vector. Then, 11–41 indicated that the strain (contains pET28a-PpBMT) was induced at 11–41°C. Markers are displayed in the left and the protein molecular weight of 97.2, 66.4, 44.3, 29, 20.1, and 14.3 kDa are listed from top to the bottom. The arrow was used to point the expressed protein in the figure. [file Image_1.TIF]

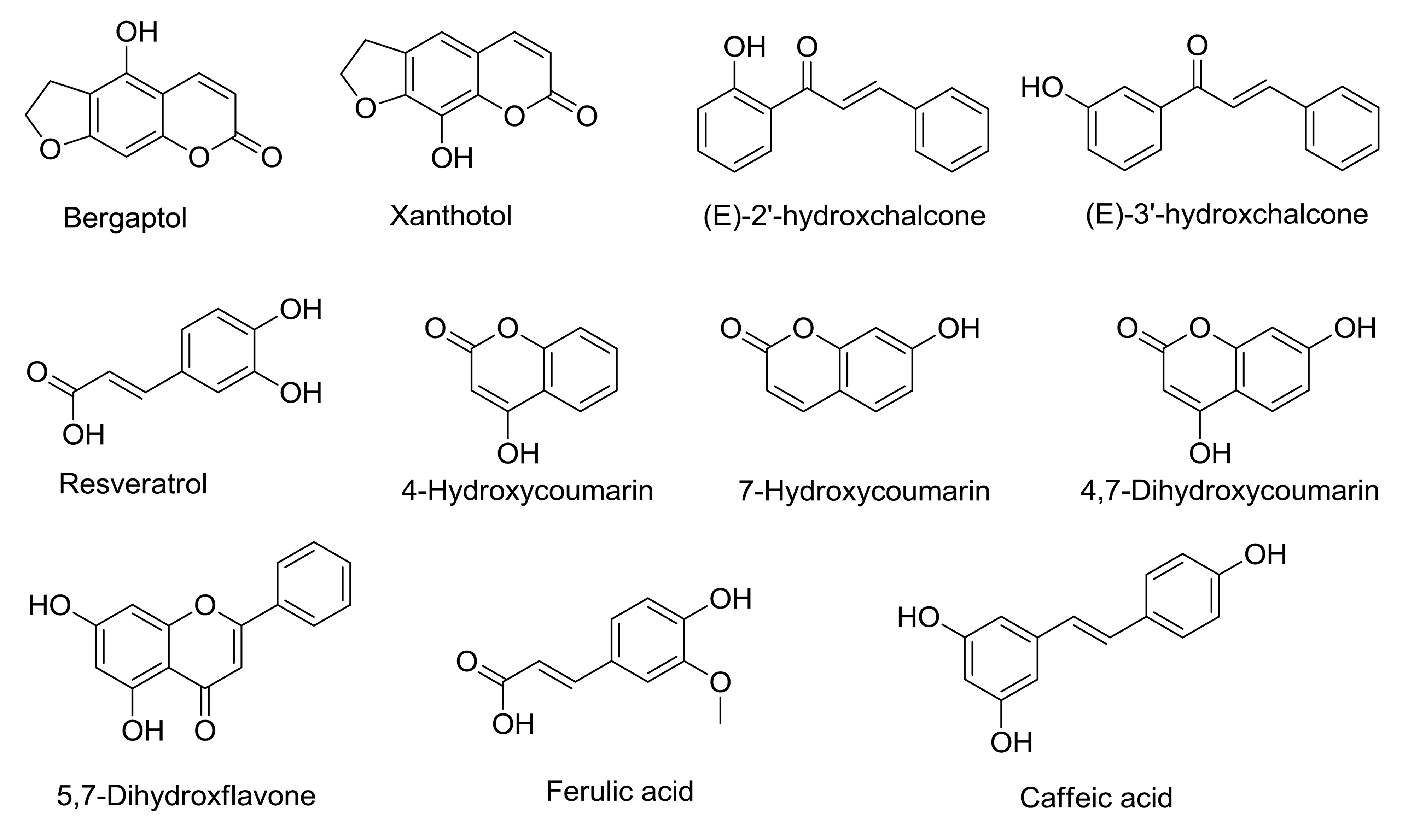

Supplement: FIGURE S2 — The different substrates used in this study. [file Image_2.TIF]

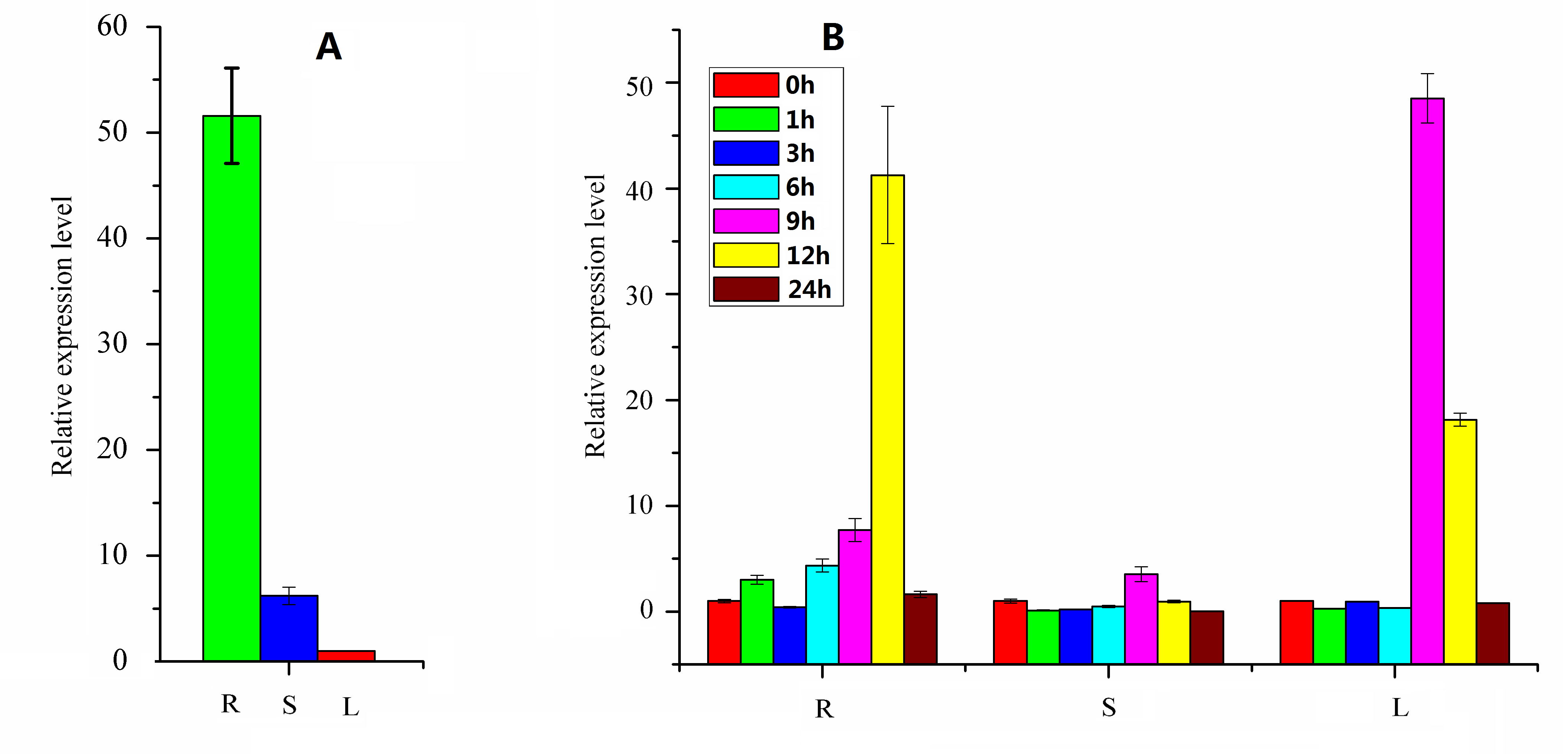

Supplement: FIGURE S3 — Expression profiles of PpBMT using another gene-specific primer. (A) Tissue-specific expression. (B) Gene expression after MeJA treatment. R, roots; S, stems; L, leaves. 0, 1, 3, 6, 9, 12, and 24 h represent the time interval after MeJA treatment. The expression level of leaves in (A) and 0 h in (B) were set as reference in each group. Each bar represents the mean value results from the mean of triplicate experiments ±SD. [file Image_3.TIF]

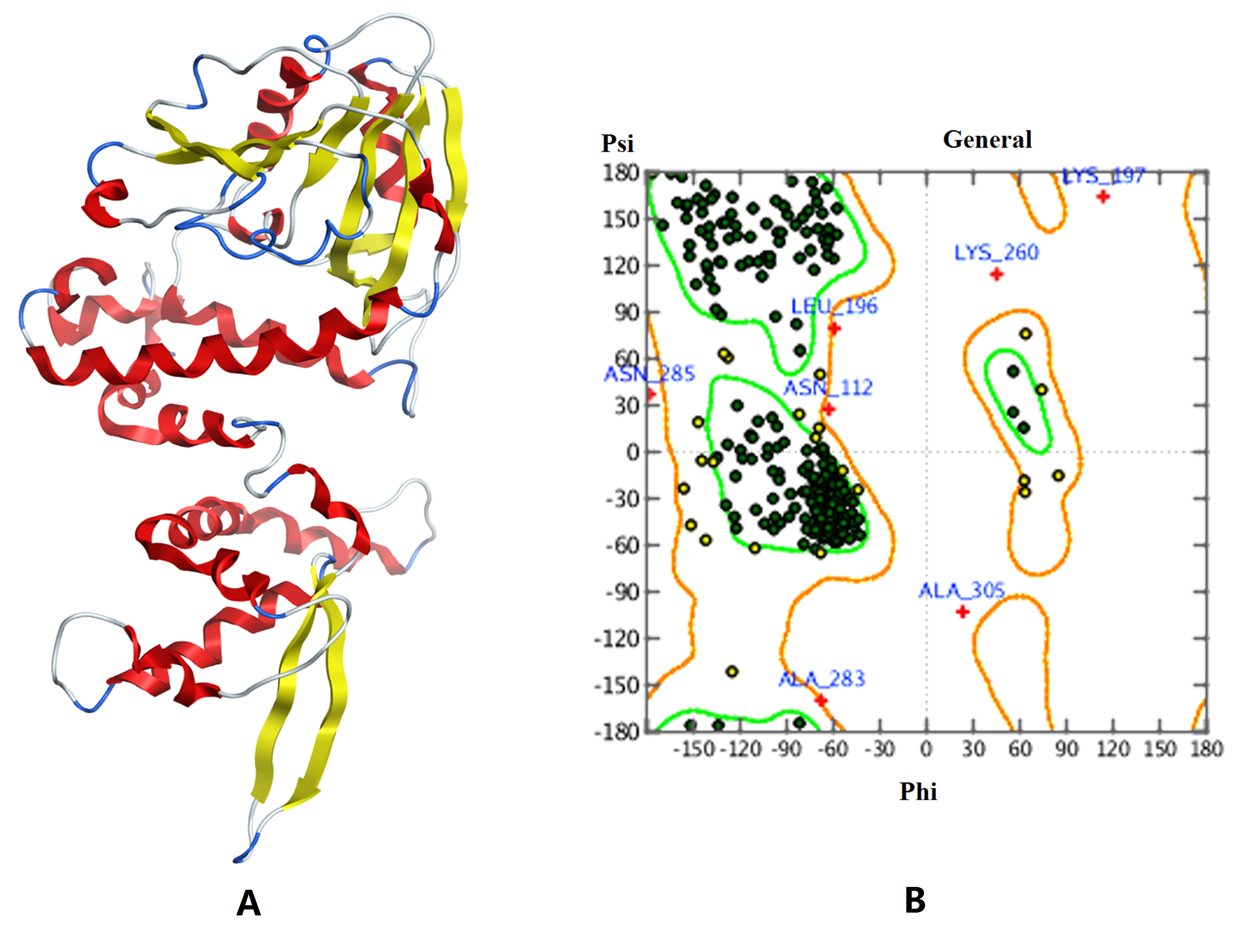

Supplement: FIGURE S4 — Homology model and docking of PpBMT. (A) The best homology model of PpBMT. (B) One of the Ramachandran plots of docking. [file Image_4.TIF]
