# Supplementary material for: Cloning, Functional Characterization, and Catalytic Mechanism of a Bergaptol O-Methyltransferase from Peucedanum praeruptorum Dunn
Source: Front Plant Sci. 2016 May 25;7:722. doi: 10.3389/fpls.2016.00722 (PMC4879325; doi:10.3389/fpls.2016.00722)
Supplement: Supplementary file 5 [file Table_1.DOCX]

**Table S1 Primers used in this study.**

| Primer name | (5'to3') |
| --- | --- |
| BMT-GSP2 | CCATTTCCAAAGCTGGCCCCGGTAA |
| BMT-GSP1 | GCGCAGCACACCTTGCGGAAACTTT |
| BMT-NGSP2 | ATGCACCCATCATGCTTGAACGCAT |
| BMT-NGSP1 | CCACCCGGAACTTCAGGAATGATGA |
| 28a-SacI-F | GAGCTCATGGCAGGAATGAAGACT |
| 28a-NotI-R | GCGGCCGCCTACTTGGAAAATTCCATAAT |
| SAND-Q-F | ACAGAAGAGCCTCATGAATCCC |
| SAND-Q-R | CAAGCAAAGGCGTCATATCAAA |
| BMT-Q-F | TTAACCGGTCAATGGCAGG |
| BMT-Q-R  BMT-Q-2-F  BMT-Q-2-R | TTAAGGGTAGCACCGCTGC  GGCATCAACTTCGACCTTCC  GATCCTCAGGCAATCTTCAT |
| 1302-NcoI-F | CCATGGCAGGAATGAAGACTAGTCC |
| 1302-BglII-R | AGATCTACCATCTTGGAAAATTCCATAATCC |
| H264A-2F | GTGGATATTTGATAGTTGGAGTGATGAAG |
| H264A-1R | ATCTTCATCACTCCAACTATCAAATATCCACTTCAAGAAT |
| Y319A-2F | AATGTTGGCAGCTGTTCCCGGGGGAAAAG |
| Y319A-1R | CTTTTCCCCCGGGAACAGCTGCCAACATT |
| M175A-2F | TTTAACCGGTCAGCGGCAGGTCATTCTACT |
| M175A-1R | AGTAGAATGACCTGCCGCTGACCGGTTAAA |
| M316A-2F | TGTGGTTCATGCTGATGCTGTAGCGTTGGCATATGTTCCC |
| M316A-1R | GGGAACATATGCCAACGCTACAGCATCAGCATGAACCACA |
| F171A-2F | CCTCAATTTAACAAGGTGGCTAACCGGTCAATGGCAGGTC |
| F171A-1R | GACCTGCCATTGACCGGTTAGCCACCTTGTTAAATTGAGG |
| L312A-2F | ACTAAGAGTGTGGTTCATGCTGATGCTGTAATGTTGGCAT |
| L312A-1R | ATGCCAACATTACAGCATCAGCATGAACCACACTCTTAGT |
| G203A-2F | AAATCTATAGTTGATGTCGCTGGTGGCAGCGGTGCTACCC |
| G203A-1R | GGGTAGCACCGCTGCCACCAGCGACATCAACTATAGATTT |
| D226A 2F | TTAAAGGCATCAACTTCGCCCTTCCTCACGTTGTGGGAGA |
| D226A 1R | TCTCCCACAACGTGAGGAAGGGCGAAGTTGATGCCTTTAA |
|  |  |
