# Supplementary material for: Cloning, Functional Characterization, and Catalytic Mechanism of a Bergaptol O-Methyltransferase from Peucedanum praeruptorum Dunn
Source: Front Plant Sci. 2016 May 25;7:722. doi: 10.3389/fpls.2016.00722 (PMC4879325; doi:10.3389/fpls.2016.00722)
Supplement: Supplementary file 6 [file Table_2.DOCX]

**Table S2 Accession numbers, the name of the species and amino acid sequences used in this study.**

PpBMT (*Peucedanum praeruptorum* bergaptol-O-methyltransferase)

MAGMKTSPSQDEEACVLAIQLATSTVLPMILKSAIELDILNTISKAGPGNYLSPSDLASKLLMSNPHAPIMLERILRVLATYKVLGCKPSELSDGEVEWLYCWTPVCKFLSNNEDGASIAPLLLVHQDQVPMKSWYHLTDAILDGGTAFNKAYGMNIFDYASQDPQFNKVFNRSMAGHSTITMKKILETYNGFEGLKSIVDVGGGSGATLNMIISKYPTIKGINFDLPHVVGDSPIHPGVEHVGGDMFASVPKGDAIFLKWIFHSWSDEDCLRILKNCYEALADNKKVIVAEFIIPEVPGGSDDATKSVVHLDAVMLAYVPGGKERTEKEFEALATSAGFKSFRKVCCAFNTWIMEFSK

AmBMT (*Ammi majus* AY443006)

MAEMKTSPSQDEEAGVVAMQLATSTVLPMILKSAIELDLLNTIAKAGPGNYLSPSDLASKLLLSNPDAPVMLARILRVLATYKVLGCKRGEVEWLYCWTPVCKYLSNNEDGASIAPILLVHQDKVTIKSWYHLTDAVRDGGTAFNKAHDMSIFEYASQDPQFNKAFNRSMRGHSTITMKKILETYKGFEGLKSIVDVGGGTGATLNMIISKYPTIKGINFDLPHVVGDAPSLPGVEHVGGNMFASVPKGDAIFLKWIFHSWGDEECLKILKKCHQALGDNKKVIVAEFILPEDPGGSDSATKSAVHLDAIMLAYVPGGKERTEKEFESLAKRAGFKSFTKVCCAFNTWIMEFSK

GlBMT (*Glehnia littoralis* AB363638)

MAGMKSSPSQDEEACVLAIQLATSTVLPMILKSAIELDILNTISKAGPGNYLSPSDLASKLLMSNPHAPIMLERILRVLATYKVLGCKRSELSNGEVEWLYCWTPVCKFLSNNEDGASIAPLLLVHQDKVPMKSWYHLTDAVLDGGTAFNKAYGMNIFDYASQDPQFNKVFNRSMAGHSTITMKKIVETYNGFEGLKSIVDVGGGSGATLNMIISKYPTIKGINFDLPHVVGDSPIHPGVEHVGGDMFASVPKGDAIFLKWIFHSWSDEDCLRILKNCYEALADNKKVIVAEFIIPEVPGGSDDATKSVVHLDAIMLAYVPGGKERTEKEFESLATRAGFKSFRKVCCAFNTWIMEFSK

AdBMT (*Angelica dahurica* AEO21927.1)

MAEMKTSPSQDEEACLLAMQLATSTVLPMILKSAIELDILNTISKAGPGNYLSPSDLASKLHISNPDARIMLGRILRVLATYKVLGCKPSELSNGKVEWLYCWTPVCKFLSNNEDGASLAPLLLGHQDKVPMKSWYHITDAVLEGGTAFNKAYGMSIFEYASQDPLFNKVFNQSMTGHSTLTMKKILETYNGFQGLKSVVDVGGGSGATLNMIISKYPTIRGINFDLPHVVGDSPIYPGVEHVGGDMFASVPKGDAIFLKWIFHSWSDEDCLRILKNCYEALADNKKVIVAEFIIPEVPDGSDGATKSVVHLDSIMLAHVPGGKERTEKEFEALATSAGFKSFSKVCCAFNTWIMEFSK

MsChOMT (*M. sativa* AAB48059)

MGNSYITKEDNQISATSEQTEDSACLSAMVLTTNLVYPAVLNAAIDLNLFEIIAKATPPGAFMSPSEIASKLPASTQHSDLPNRLDRMLRLLASYSVLTSTTRTIEDGGAERVYGLSMVGKYLVPDESRGYLASFTTFLCYPALLQVWMNFKEAVVDEDIDLFKNVHGVTKYEFMGKDKKMNQIFNKSMVDVCATEMKRMLEIYTGFEGISTLVDVGGGSGRNLELIISKYPLIKGINFDLPQVIENAPPLSGIEHVGGDMFASVPQGDAMILKAVCHNWSDEKCIEFLSNCHKALSPNGKVIIVEFILPEEPNTSEESKLVSTLDNLMFITVGGRERTEKQYEKLSKLSGFSKFQVACRAFNSLGVMEFYK

MsCOMT (*M. sativa* AAB46623)

MGSTGETQITPTHISDEEANLFAMQLASASVLPMILKSALELDLLEIIAKAGPGAQISPIEIASQLPTTNPDAPVMLDRMLRLLACYIILTCSVRTQQDGKVQRLYGLATVAKYLVKNEDGVSISALNLMNQDKVLMESWYHLKDAVLDGGIPFNKAYGMTAFEYHGTDPRFNKVFNKGMSDHSTITMKKILETYTGFEGLKSLVDVGGGTGAVINTIVSKYPTIKGINFDLPHVIEDAPSYPGVEHVGGDMFVSIPKADAVFMKWICHDWSDEHCLKFLKNCYEALPDNGKVIVAECILPVAPDSSLATKGVVHIDVIMLAHNPGGKERTQKEFEDLAKGAGFQGFKVHCNAFNTYIMEFLKKV

HvOMT (*Hordeum vulgare* CAA54616)

MQDTSSTQHKSLPNNIIEMDMVTSMPLEANSNGQILQAEAELFCHSFGYLKSMALQSVVKLRIPDVLHRYGGAASLPELLSTVPIHPNKLPYLPRLMKMLAAAGIFTAEDVPATVGDGEPTTLYHLNAVSRLLVDDASVNGGASMSPCVLLGTVPLFLGASLKLHEWLQSEEQATTETPFMLAHGGTLYGIGGRDSEFNTVFNKAMGASSEFVAALAVRECRDVFAGIKSLVDVAGGNGTTARTIAEAFPYVKCSVLDLPQVIQGISSHGTVEFVAGDMMEFVPPAEAVLLKYVLHNWSDQDCVKILTRCREAISHGEKAGKVIIIDTVVGSPSQQILESQVTMDLSMMMLFNGKVREEQNWHKIFLEAGFSHYKIHNVLGMRSLIEVQP

AtOMT (*Arabidopsis* NP_195242.1)

MSSDQLSKFLDRNKMEDNKRKVLDEEAKASLDIWKYVFGFADIAAAKCAIDLKIPEAIENHPSSQPVTLAELSSAVSASPSHLRRIMRFLVHQGIFKEIPTKDGLATGYVNTPLSRRLMITRRDGKSLAPFVLFETTPEMLAPWLRLSSVVSSPVNGSTPPPFDAVHGKDVWSFAQDNPFLSDMINEAMACDARRVVPRVAGACHGLFDGVTTMVDVGGGTGETMGMLVKEFPWIKGFNFDLPHVIEVAEVLDGVENVEGDMFDSIPACDAIFIKWVLHDWGDKDCIKILKNCKEAVPPNIGKVLIVESVIGENKKTMIVDERDEKLEHVRLMLDMVMMAHTSTGKERTLKEWDFVLKEAGFARYEVRDIDDVQSLIIAYRS

CaOMT (*Capsicum annuum* Q9FQY8.2)

MDSTNQNLTQTEDEAFLFAMQLASASVLPMVLKSALELDLLEIMAKAGPGAAISPSELAAQLPTKNPEAPVMLDRMLRLLATYSVLNCTLRTLPDGRVERLYSLAPVCKLLTKNADGVSVAPLLLMNQDKVLMESWYHLTDAVLDGGVPFNKAYGMTAFEYHGTDPRFNKVFNRGMSDHSTMTMKKILEDYKGFEGLNSIVDVGGGTGATVNMIVSKYPSIKGINFDLSHVIEDAPAYPGVEHVGRDMFVSVPKADAIFMKWICHDWSDEHCLKFLKNCYEALPANGKVLVAECILPETPDTSAATKNAVHVDIVMLAHNPGGKERTEKEFEALAKGAGFTGFRRACCAYQTWVMEFHK

NtOMT (*Nicotiana tabacum* CAA52461.1)

MGSTSESQSNSLTHTEDEAFLFAMQLCSASVLPMVLKSAVELDLLELMAKAGPGAAISPSELAAQLSTQNPEAPVMLDRMLRLLASYSVLNCTLRTLPDSSVERLYSLAPVCKYLTKNADGVSVAPLLLMNQDKVLMESWYHLKDAVLDGGIPFNKAYGMTAFEYHGTDPRFNKVFNRGMSDHSTMSMKKILEDYKGFEGLNSIVDVGGGTGATVNMIVSKYPSIKGINFDLPHVIGDAPTYPGVEHVGGDMFASVPKADAIFMKWICHDWSDEHCLKFLKNCYEALPANGKVIIAECILPEAPDTSLATKNTVHVDIVMLAHNPGGKERTEKEFEALAKGAGFTGFARLVALTTLGSWNSTSN

CaFOMT (*Chrysosplenium americanum* Q42653.1)

MLFAMQLACASVLPMVLKSAIELDLLEIIRGQDTCMSPTEIASHLPTTNPDAPAMVDRILRLLSCYSVVTCSVRSVDDQRVYGLAPVCKYLTKNQDGVSIAALCLMNQDKVLMESWYHLKDAVLDGGIPFNKAYGMSSFEYHGTDPRFNKVFNRGMSDHSTITMKKVFQTYQGFQGLTSLVDVGGGTGATLTMILSKYPTIRCINFDLPHVIEDAPEYPGIEHVGGDMFVSVPKGDAIFMKWICHDWSDEHCLKLLKNCYDALPNNGKVILAECILPEVPDSSLATKGVVHIDVITVAHNPGGKERTEKEFEALAKAAGFQGFQVFCNAFNTYIIEFSKKICN

CbIEMT (*Clarkia breweri* O04385.2)

MGSTGNAEIQIIPTHSSDEEANLFAMQLASAAVLPMALKAAIELDVLEIMAKSVPPSGYISPAEIAAQLPTTNPEAPVMLDRVLRLLASYSVVTYTLRELPSGKVERLYGLAPVCKFLTKNEDGVSLAPFLLTATDKVLLEPWFYLKDAILEGGIPFNKAYGMNEFDYHGTDHRFNKVFNKGMSSNSTITMKKILEMYNGFEGLTTIVDVGGGTGAVASMIVAKYPSINAINFDLPHVIQDAPAFSGVEHLGGDMFDGVPKGDAIFIKWICHDWSDEHCLKLLKNCYAALPDHGKVIVAEYILPPSPDPSIATKVVIHTDALMLAYNPGGKERTEKEFQALAMASGFRGFKVASCAFNTYVMEFLKTA

EgCOMT (*Eucalyptus gunnii* P46484.1)

MGSTGSETQMTPTQVSDEEANLFAMQLASASVLPMVLKAAIELDLLEIMAKAGPGAFLSPGEVAAQLPTQNPEAPVMLDRIFRLLASYSVLTCTLRNLPDGKVERLYGLAPVCKFLVKNEDGVSIAALNLMNQDKILMESWYYLKDAVLEGGIPFNKAYGMTAFEYHGTDPRFNKIFNRGMSDHSTITMKKILETYKGFEGLETVVDVGGGTGAVLSMIVAKYPSMKGINFDLPHVIEDAPPLPGVKHVGGDMFVSVPKGDAIFMKWICHDWSDDHCAKFLKNCYDALPNIGKVIVAECVLPVYPDTSLATKNVIHIDCIMLAHNPGGKERTQKEFETLAKGAGFQGFQVMCCAFGTHVMEFLKTA

CjOMT (*Coptis japonica* Q39522.1)

MCTSLSELKCPVFSTKRKLLLEFALRTSVDMAAQEGVNYLSGLGLSRLICLPMALRAAIELNVFEIISQAGPDAQLSPSDIVAKIPTKNPSAAISLDRILRMLGASSILSVSTTKSGRVYGLNEESRCLVASEDKVSVVPMLLFTSDKAVVESFYNIKDVVLEEGVIPFDRTHGMDFFQYAGKEERVNKSFNQAMGAGSTIAFDEVFKVYKGFDNLKELVDVGGGIGTSLSNIVAKHPHIRGINFELPHVIGDAPDYPGVEHVPGDMFEGVPNAQNILLKWVLHDWDDDRSIKILKNCWKALPENGTVIVIEFVLPQVLGNNAESFNALTPDLLMMALNPGGKERTTIEFDGLAKAAGFAETKFFPISQGLHVMEFHKINC

PsOMT (*Pisum sativum* O24305.1)

MDFSTNGSEESELYHAQIHLYKHVYNFVSSMALKSAMELGIADAIHNHGKPMTLPELSSSLKLHPSKVNILYRFLRLLTHNGFFAKTTVKSNEGEEETAYVLTPSSKLLVSGKSTCLSSLVKGALHPSSLDMWGVSKKWFHEDKEQTLFECATGENYWDFLNKDSDSLSMFQDAMAADSRLFKLAIQENKHVFEGLESLVDVAGGTGGVAKLIHEAFPHIKCTVFDQPQVVGNLTGNENLNFVGGDMFKSVPSADAVLLKWVLHDWNDELSLKILKNSKEAISHKGKDGKVIIIDISIDENSDDRGLTELQLEYDVVMLTMFLGKERTKKEWEKLIYDAGFSRYKITPICGFKSLIEVYP

PdCOMT (*Prunus dulcis* Q43609.1)

MGSTGETQMTPTQVSDEEANLFAMQLASASVLPMVLKAAIELDLLEIMAKAGPGVFLSPTDIASQLPTKNPDAPVMLDRMLRLLASYSILTYSLRTLADGKVERLYGLGPVCKFLTKNEEGVSIAPLCLMNQDKVLLESWYHLKDAVLEGGIPFNKAYGMTAFEYHGTDPRFNKVFNRGMADHSTITMKKILETYKGFEGLTSVVDVGGGTGAVLNMIVSKYPSIKGINFDLPHVIEDAPQYPGVEHVGGDMFVSVPKGDAIFMKWICHDWSDEHCLKFLKNCYAALPDNGKVILGECILPVAPDSSLATKGVVHIDVIMLAHNPGGKERTEQEFQALAKGAGFQGFNVACSAFNTYVIEFLKKN

PpOMT (*Pyrus pyrifolia* BAA86059.1)

MSSSNAVGATSHELLGAQAQLWNHIFQFINSMSLKCAVQLGIADVIHNHGQPISLSELIAGLNVHPSKAHFVSRLMLILVHSNFFAQHHHVHHDRADVEEEEAVVLYSLTPSSRLLLKDGPFSTTPFLLATLDPVVTTPFHLMGAWLKINGGDDPGATCTPFEMENGMPFWELGAQEPRFGNLFNEAMEADSKLIGRVVVEECGGVFEGLKSLMDVGGGSGTMAKAIANAFPNINCTVFDQPHVVAGLQGTTHNLGFMGGDMFEEIPPANAILLKWIMHDWNDEESVTILKKCREAISLSKNEGGNKKIIIIDIVVGYVDNKKKMMDKKSIETQLMFDMLMMSILPGKERSKLEWEKIFFSAGFTHYNITHTLGLRSLIEVYP

MsIOMT (*M. sativa* O22309.1)

MASSINGRKPSEIFKAQALLYKHIYAFIDSMSLKWAVGMNIPNIIHNHGKPISLSNLVSILQVPSSKIGNVRRLMRYLAHNGFFEIITKEEESYALTVASELLVRGSDLCLAPMVECVLDPTLSGSYHELKKWIYEEDLTLFGVTLGSGFWDFLDKNPEYNTSFNDAMASDSKLINLALRDCDFVFDGLESIVDVGGGTGTTAKIICETFPKLKCIVFDRPQVVENLSGSNNLTYVGGDMFTSIPNADAVLLKYILHNWTDKDCLRILKKCKEAVTNDGKRGKVTIIDMVINEKKDENQVTQIKLLMDVNMACLNGKERNEEEWKKLFIEAGFQHYKISPLTGFLSLIEIYP

ObCOMT (*Ocimum basilicum* Q9XGW0.1)

MGSATNTPQINSDEEENFLFAMQLASASVLPMVLKSAIELDLLELIKKSGAGAFVSPVDLAAQLPTTNPDAHVMLDRILRLLTSYAILECRLKTLPDGGVERLYGLAPVCKFLTKNEDGVSMAPLTLMNQDKVLMESWYHLSDAVVDGGIPFNKAYGMTAFEYHGTDPRFNKVFNQGMSNHSTITMKKILETYTGFDGLKTVVDVGGGTGATLNMIVSKYPSIKGINFDLPHVIEDAPSYPGVEHVGGDMFVSVPKGDAIFMKWICHDWSDEHCVKFLKNCYDALPQNGKVILAECVLPEAPDTGLATKNVVHIDVIMLAHNPGGKERTEKEFQGLAKAAGFKQFNKACCAYNTWIMELLK

PrOMT (*Pinus radiata* AAD24001.1)

MDSNMNGLAKSNGCEISRDGFFESEEEELQGQAEAWKCTFAFAESLAVKCVVLLGIPDMIAREGPRATLSLGEIVAKLPTESPDAACLFRIMRFLVAKGIFRASKSAREGGAFETRYGLTPASKWLVKGRELSMAPMLLMQNDETTLAPWHHFNECVLEGGVAFQKANGAEIWSYASDHPDFNNLFNNAMACNARIVMKAILSKYQGFHSLNSLVDVGGGTGTAVAEIVRAYPFIRGINYDLPHVVATASSLSGVQHVGGDMFETVPTGDAIFMKWIMHDWNDEDCIKILKNCRKAIPDTGKVIIVDVVLDADQGDNTDKKRKKAVDPIVGTVFDLVMVAHSSGGKERSEKEWKRILLEGGFSRYNIIEIPALQSVIEAFPR

PaOMT (*Prunus armeniaca* AAB71213.1)

MGSVRASHELLQAQAHIWNHIFSFINSLSLKCAVQLDIPDVIQKHGQPMTLSELVSALPISPTKAHFIPRLMRILVHSGFFAKESLSGCGEQGYILTDASALLLKDNPMSARPFLLAMLSPILTDPYQYLTTWFQNDNPTPFHVVNGMTCWDYVNQDPTLAHFFNDAMASDAQLISSLVIDDCKEVFQGVDSLVDVGGGTGTVAKSIADAFPHMKCTVLDLPHVVADLKGSKNLEYVAGDMFEAVPAADAIFLKWILHDWSDEECVKILERCKAAVTREGKKGKVIIVEMTVENKNTDKESGETQLFFDMHMMVMSTGKERNEKEWAKLFSDAGFSQYKITPLFGFKSLIEVYP

TaOMT (*Triticum aestivum* AAD10485.1)

MALTGDYKLISTDDMLQGHAELCIHAYGFVKSMALKCAIELGIPGAIHGHGGGATLGELATIIALPPSRLPRLRRLMRVLTVSGVFSVQNQQPDDPAGCAAVVYGLTAASRLLVGDGESIVGAAPPRSLMVDPNLTAPFSGMSAWFMDDEQPRSFFEMHHGEDMWEMAARDAALSRTIGDGMTDDSRFVVEVLLREGRARDVFSGVRSMVDVGGGTGTIAKAIAAAFPHVECSVLDLPHVVAEAPAGGEVRFIEGDMFEHIPPADAVLLKSVMHDWRDDECVKILRRCKEAIPSREAGGKVIIINMVVGSEKSKGNSTKKGGGTSIVRSSSSWFLREVNEKSMSGRRSFLEAGFSGYSIIPMLGIRSIIEVYP

ZvCOMT (*Zinnia violacea* Q43239.1)

MGSNQDDQAFLFAMQLASASVLPMVLKTAIELDLLETIAKAGPHGSVSSSELVAQLPKVNNPEAPVMIDRICSLLASYSVLTCTLKETADGCAERFYGLAPVCKFLIKNDAGVSLAPLLLMNQDKVLMESWYYLKDPVLDGGIPFNKAYGMSAFEYHGKDQRFNKVFNSGMFNHSTMTMKKIVELYNGFSGLKTLVDVGGGTGASLNMITSKHKSLKGINFDLPHVIADATTYQGIEHVGGDMFESVPKGDAIFMKWILHDWSDAHCLQVLKNCYKSLPENGKVIVAECILPEAPDTTPATQNVIHIDVIMLAHNPGGKERTEKEFEALAKGAGFKGFNKAACALNTWVMEFCK
