# Supplementary material for: Cloning, Functional Characterization, and Catalytic Mechanism of a Bergaptol O-Methyltransferase from Peucedanum praeruptorum Dunn
Source: Front Plant Sci. 2016 May 25;7:722. doi: 10.3389/fpls.2016.00722 (PMC4879325; doi:10.3389/fpls.2016.00722)
Supplement: Supplementary file 7 [file Table_3.DOCX]

**Table S3 The nucleotide and protein sequence of PpBMT**

(a) Nucleotide sequence of PpBMT. The 13bp 5’ and 263 bp 3’ untranslated regions are in yellow. The initiation codon (atg) and termination codon (tag) are in red. (b) Protein sequence of PpBMT.

(a) Nucleotide sequence

acatggggattcaatggcaggaatgaagactagtccatctcaagacgaagaagcttgtgtgctagccattcaattagcaacctctacggtgcttcccatgattctcaaatcagcaatagagcttgacatactaaataccatttccaaagctggccccggtaactatttaagtccttctgatctagcctctaagcttctcatgtcgaacccccatgcacccatcatgcttgaacgcatcctccgagtcctggctacctacaaagttcttggttgtaagcctagtgaactttccgacggcgaagttgagtggctctactgctggacacccgtgtgcaagttcttgtccaataacgaagacggtgcttctatagcaccgcttttgttagtgcaccaggaccaagttccaatgaagagttggtatcatctaacagatgcaattctggatggaggaactgcatttaacaaggcctatggaatgaatatattcgattatgctagccaagatcctcaatttaacaaggtgtttaaccggtcaatggcaggtcattctactataaccatgaaaaaaatccttgaaacttacaatggtttcgaaggtctcaaatctatagttgatgtcggtggtggcagcggtgctacccttaatatgatcatctccaagtaccctactattaaaggcatcaacttcgaccttcctcacgttgtgggagactctccaattcatcctggtgtggaacatgtagggggagacatgttcgctagtgtgccaaaaggagacgccatattcttgaagtggatatttcatagttggagtgatgaagattgcctgaggatcttgaaaaattgttacgaagctttggcggataataagaaggtcatcgttgcagaattcatcattcctgaagttccgggtggtagcgacgatgcaactaagagtgtggttcatcttgatgctgtaatgttggcatatgttcccgggggaaaagagaggacagaaaaagagtttgaagctttagctacaagtgcaggatttaaaagtttccgcaaggtgtgctgcgctttcaatacttggattatggaattttccaagtagctagagtgctctgtgttcgaaagtgtttttatgagtacttctctttcgtaatcccagctccaaatatgcaataagggccacaatcccaaacgtgtaactctaatttatttcaaagtccgaaatgtgttttctttgtacaattgtactctttgctttctaaaaataggaagggagattgttccatgtccgatttcaaataattgagcagcagttccgttggcaagaattgtggctaaaaaaaaaaaaaaaaaaaaaaaaaaaaa

(b) Protein sequence:

MAGMKTSPSQDEEACVLAIQLATSTVLPMILKSAIELDILNTISKAGPGNYLSPSDLASKLLMSNPHAPIMLERILRVLATYKVLGCKPSELSDGEVEWLYCWTPVCKFLSNNEDGASIAPLLLVHQDQVPMKSWYHLTDAILDGGTAFNKAYGMNIFDYASQDPQFNKVFNRSMAGHSTITMKKILETYNGFEGLKSIVDVGGGSGATLNMIISKYPTIKGINFDLPHVVGDSPIHPGVEHVGGDMFASVPKGDAIFLKWIFHSWSDEDCLRILKNCYEALADNKKVIVAEFIIPEVPGGSDDATKSVVHLDAVMLAYVPGGKERTEKEFEALATSAGFKSFRKVCCAFNTWIMEFSK
